# Differences between serum polar lipid profiles of male and female rheumatoid arthritis patients in response to glucocorticoid treatment

**DOI:** 10.1007/s10787-016-0284-1

**Published:** 2016-09-28

**Authors:** Junzeng Fu, Bart V. J. Cuppen, Paco M. J. Welsing, Herman van Wietmarschen, Amy C. Harms, Ruud Berger, Slavik Koval, Ruth D. E. Fritsch-Stork, Johannes W. J. Bijlsma, Thomas Hankemeier, Jan van der Greef, Floris P. J. G. Lafeber

**Affiliations:** 1 Leiden Academic Center for Drug Research, Leiden University, Einsteinweg 55, 2333 CC Leiden, The Netherlands; 2 Rheumatology and Clinical Immunology, University Medical Center Utrecht, F02.127, Heidelberglaan 100, 3584 CX Utrecht, The Netherlands; 3 Sino-Dutch Center for Preventive and Personalized Medicine, P.O. Box 360, 3700 AJ Zeist, The Netherlands; 4 TNO, Netherlands Organization for Applied Scientific Research, Microbiology and Systems Biology, Zeist, The Netherlands; 5 Netherlands Metabolomics Center, Einsteinweg 55, 2333 CC Leiden, The Netherlands; 6 1st Medical Department and Ludwig Boltzmann Institute of Osteology at the Hanusch Hospital of WGKK and AUVA Trauma Centre Meidling, Hanusch Hospital, Heinrich-Collin-Straße 30, 1140 Vienna, Austria; 7 Sigmund Freud University, Freudplatz 1, 1020 Vienna, Austria

**Keywords:** Gender difference, Lysophospholipid, Glucocorticoid, Rheumatoid arthritis

## Abstract

**Objective:**

As there are pharmacological differences between males and females, and glucocorticoid (GC) treatment is associated with increased cardiovascular mortality rate in rheumatoid arthritis (RA) patients, it is important to study serum polar lipid profiles of male and female patients in response to GC therapy. Gender differences may require an adjustment to the treatment strategy for a selection of patients.

**Methods:**

Serum samples from 281 RA patients were analysed using a targeted lipidomics platform. The differences in GC use and gender on polar lipid profiles were cross sectionally examined by multiple linear regressions, while correcting for confounding factors.

**Results:**

Differences in polar lipids between GC users and non-GC users in females and males were merely restricted to lysophospholipids (lysophosphatidylcholines and lysophosphatidylethanolamines). Lysophospholipids in female patients treated with GCs were significantly higher than female patients not treated with GCs (*p* = 6.0 E−6), whereas no significant difference was observed in male GC users versus non-users (*p* = 0.397).

**Conclusion:**

The lysophospholipid profiles in response to GCs were significantly different between male and female RA patients, which may have implications for the cardiovascular risk of GC treatment.

**Electronic supplementary material:**

The online version of this article (doi:10.1007/s10787-016-0284-1) contains supplementary material, which is available to authorized users.

## Introduction

Rheumatoid arthritis (RA) is an auto-immune disease with unresolved aetiology which predominantly occurs in females (Jutley et al. 2015). Glucocorticoids (GCs) have been prescribed for the treatment of RA for decades, and are considered to be effective drugs in reducing inflammation and preventing joint destruction (Hoes et al. 2007). The role of gender as a crucial factor in drug studies is becoming increasingly appreciated (Soldin and Mattison 2009). Several studies have investigated the effects of gender on clinical pharmacology for GCs on healthy volunteers, and showed gender specific differences in GC pharmacokinetics and pharmacodynamics (Lew et al. 1993; Magee et al. 2001). However, these differences are balanced by complementary GC clearance and GC sensitivity. Therefore, these gender differences do not necessitate GC dose adjustments in clinical practice.

Glucocorticoids are known to undesirably affect triacylglyceride and fatty acid metabolism (Macfarlane et al. 2008). It is thus conceivable that GC-induced changes of lipid profiles in RA patients also show gender dependence, apart from the gender differences in lipid metabolism seen in the general population (Wang et al. 2011). Because GC treatment is associated with increased cardiovascular mortality rate in RA patients (del Rincón et al. 2014; McGrath and Young 2015), it is important to study these gender-based lipid differences as they may require an adjustment in treatment strategy for a selection of the patients.

In this study, we measured circulating polar lipids, such as lysophospholipids and free fatty acids, in the serum of RA patients using a targeted lipidomics platform, because polar lipids are crucial intermediates in lipid metabolism. Lipid profiles between GC users and non-GC users were examined and analysed for gender differences. Our results suggested that the lipid profile is more affected by GC treatment in female RA patients. In particular, the levels of lysophospholipids were more elevated in female users compared to non-users. In males, the differences of lysophospholipid levels in GC users were not significant compared to non-users.

## Methods and materials

Subjects in this study were participants of an observational study BiOCURA, in which RA patients initiating or switching from biological therapy were recruited (Cuppen et al. 2016). Blood samples were collected before initiating the biological therapy and immediately processed into serum. Serum samples were stored at −80 °C until use for lipidomic analyses. At the time of sampling, 42.35 % of patients were receiving GCs, including prednisolone and prednisone, at varying dosages (all medications are listed in Supplementary Table S1). The study was approved by the medical ethics committee of the University Medical Center Utrecht and the institutional review boards of the participating centers (see Acknowledgements). Written informed consent was obtained from each patient.

The operating procedures of the targeted lipidomics platform are optimised from the previously published method (Hu et al. 2008). Polar lipids are extracted by methanol from serum samples, and analysed by liquid chromatography–mass spectrometry, covering the low abundance lipid species, including free fatty acids and lysophospholipids—lysophosphatidylcholines (LPCs) and lysophosphatidylethanolamines (LPEs). Details of the procedures are described in the Supplementary Method.

A schematic overview of the statistical analyses is provided in Supplementary Fig. S1. Initially, the obtained lipid data set was log-transformed and standardised into *Z* scores to produce normalised and auto-scaled data (mean = 0, SD = 1). Then, the differences in lipids between GC users and GC non-users were calculated and tested for significance by independent *t* tests for male and female subjects separately. In parallel, principal component analysis (PCA) was performed on all detected lipids to elucidate the correlation structure of the metabolites. By combining the results of the *t* tests, PCA, and prior biological knowledge, a decision was made on which lipids can be clustered into a new lipid score to have one overall outcome for subsequent analyses. For each patient, the score was computed by summing the standardized values of lipids and dividing this by the number of included lipids ($\sum\nolimits_{i}^{N} {\text{Standardised lipid}}$_*i*_/*N*, with *N* = number of lipids clustered). This value represents both the average of lipids, as well as the patients’ relative deviation from the mean lipid score in standard deviations (SDs). Multiple linear regression analysis was then conducted to study the effect of GC use between males and females on the lipid score. We entered the following subgroups in the model—female GC user (*n* = 77), female GC non-user (*n* = 136), male GC user (*n* = 42), and male GC non-user (*n* = 26) together with the clinical parameters (listed in Supplementary Table S1) as a full model, while setting the subgroup “female GC non-user” as the reference group (i.e., the intercept of the regression model). To arrive at a final model, backward elimination was applied on the full model by excluding clinical parameters one by one on *p* values (starting from highest to lowest *p* value). Parameters were excluded only when the change in the regression coefficients after exclusion was <10 % for all four subgroups; otherwise, the clinical parameter was kept in the model as a confounder. To explore the difference in lipid score between GC users and non-users in males, the reference group in the final model was switched to “male GC non-users”.

## Results and discussion

In the patient cohort (*n* = 281), there were more males taking GCs than females (61.8 versus 35.8 %, *p* < 0.01). There were no significant differences in disease activity among the relevant groups (Supplementary Table S1). However, the number of smokers was especially high in males (*p* < 0.001), positive rheumatoid factor was high amongst male GC users (*p* = 0.004), GC users used less non-steroidal anti-inflammatory drugs (NSAIDs) (*p* = 0.010) and more bisphosphonates (to reduce the risk of GC-induced osteoporosis) (*p* < 0.001).

**Fig. 1:**
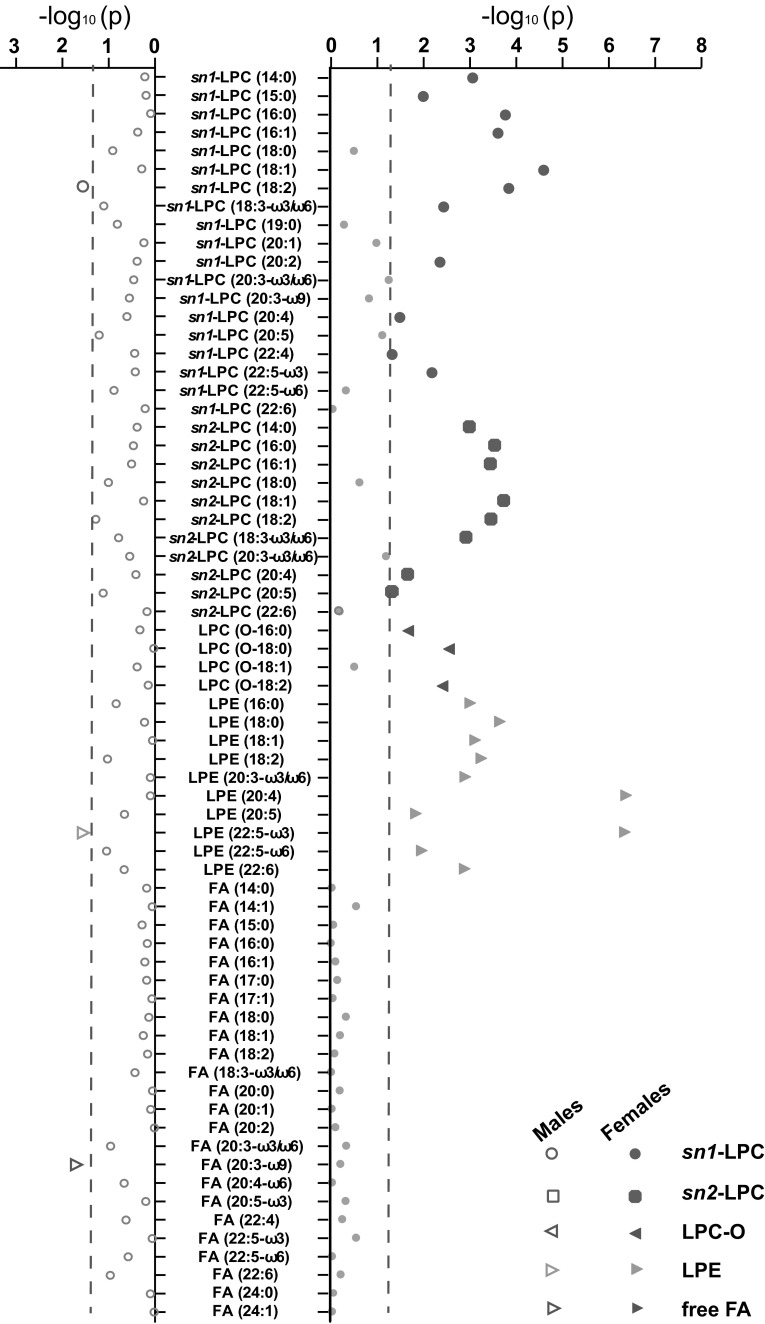
Graphical representation of *t* tests results in lipid clustering. Independent sample *t* tests were performed on all 68 metabolites on glucocorticoid (GC) users versus non-users, for both genders separately. The *x*-axis shows the logarithm of *p* values per metabolite for males and females; the blue dash line represents *p* value of 0.05

By applying an established lipidomics platform, serum lipid profiles of the 281 RA patients were analysed, covering 44 lysophospholipids and 24 free fatty acids (Supplementary Table S2). *T* tests between GC users and non-users were performed (Supplementary Table S3; a graphical representation is shown in Fig. 1). In females, we identified 10 LPEs and 22 LPCs which were significantly higher in GC users than in GC non-users (*p* < 0.05), whereas no differences were found in fatty acids. However, in male subjects, only one LPE (LPE (20:5), *p* = 0.029) and LPC (*sn1*-LPC (18:2), *p* = 0.027), which were significant in females, were found significantly higher in GC users, whereas one fatty acid (FA (20:3-ω9), *p* = 0.021) was significant lower in male GC users among all measured FAs. These results suggest that the GCs have a more pronounced impact on female lysophospholipid profile compared to males.

**Fig. 2:**
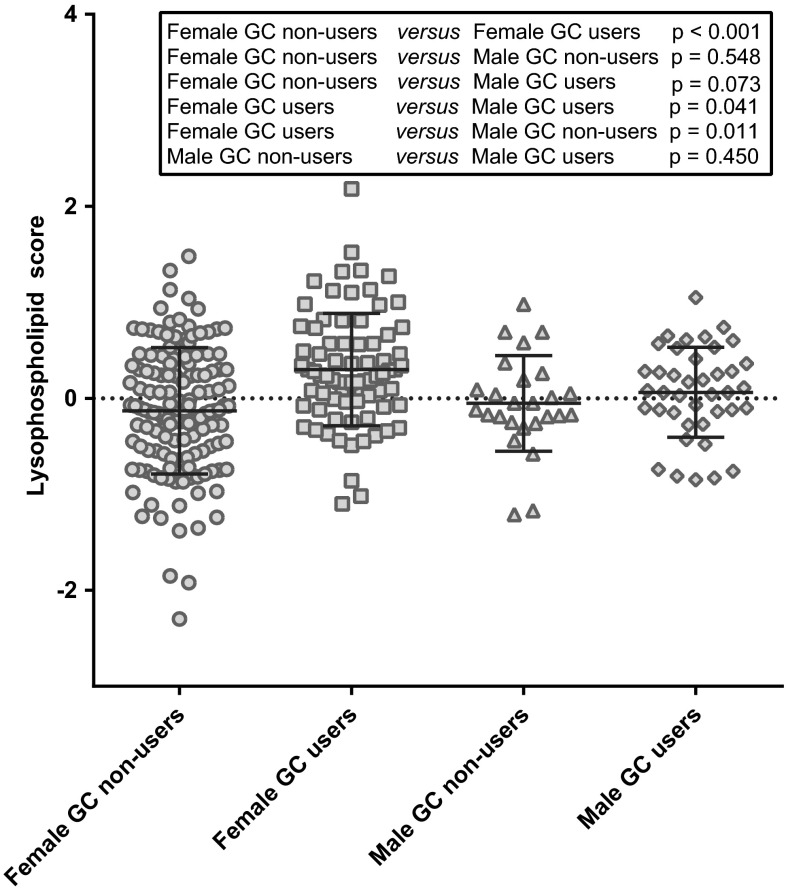
Lyophospholipid score in male patients without (*n* = 26) or with glucocorticoid (GC) treatment (*n* = 42), and in female patients without (*n* = 136) or with GC treatment (*n* = 77). Horizontal bars indicate mean values and standard deviation. One-way ANOVA with Fisher’s LSD was used to compare the means of score among subgroups

The parallel PCA analysis on 68 metabolites showed that the loading scores of all 32 significant lysophospholipids were larger than 0.4 in the first component, thus highly correlated with each other (Supplementary Fig. S2, Table S4). A new score representing the lysophospholipid levels could, therefore, be computed by calculation of the mean of all significant lysophospholipids. As shown in Fig. 2, the absolute lysophospholipid scores were significantly different between female GC users and non-users (*p* < 0.001), whereas no difference was seen in males (*p* = 0.450). In addition, female GC users showed significantly higher values compared to male GC users (*p* = 0.041), whereas no difference was seen between female and male non-users (*p* = 0.548).

After backward elimination for potential confounders, a final model for the lysophospholipid score was established (Table 1). Subgroups of males and female GC users all had significant positive coefficients, which suggest that the lysophospholipid score of these subgroups was significantly higher compared to females not using GCs. In particular, females using GCs had a significant increase in mean lysophospholipid score of 0.398 (*p* = 6E−6), compared to females not using GCs. Interestingly, the difference between male and female non-users was not significant before correcting for confounders (*p* = 0.548, Fig. 2), while it was significant in the final model (coefficient = 0.310, *p* = 0.041). The difference between GC users and non-users in males was non-significant (0.122, *p* = 0.397; model with “male GC non-users” as a reference shown in Supplementary Table S5). Therefore, after correcting for confounders, the lysophospholipids score of female RA patients taking GCs was still significantly higher than the female patients not taking GCs, whereas in male RA patients, no difference in lysophospholipid score was seen. This was consistent with the results of the *t* tests on individual lipids and the uncorrected difference in lysophospholipid score.

**Table 1:** Final regression model investigating the association between gender and glucocorticoid (GC) use on the lysophospholipid score corrected for confounders. Shown is the difference in mean lysophospholipid score for subgroups compared to females not using GCs (as reference group)

Variables	Coefficients^a^ (95 %-CI)	*p* value
Female GC non-user (reference group)	−0.161 (−0.729 to 0.408)	0.580
Female GC user	0.398 (0.229 to 0.567)	6.0 E−6
Male GC non-user	0.310 (0.015 to 0.604)	0.041
Male GC user	0.432 (0.164 to 0.700)	1.7 E−3

*BMI* body mass index, *CI* confidence interval, *CRP* C-reactive protein, *GC* glucocorticoid, *RF* rheumatoid factor, *ACPA* anti-citrullinated protein antibody, *ESR* erythrocyte sedimentation rate, *DAS28* disease activity score based on a 28-joint count, *NSAIDs* non-steroid anti-inflammatory drugs

^a^Coefficients indicate the changes in mean lysophospholipid score, adjusting for: age, BMI, menopausal status, RF positive, log-transformed CRP, log-transformed ESR, DAS28, and concomitant drugs (methotrexate, hydroxychloroquine, anti-diabetic drug and NSAIDs)

Patients with RA already have a higher cardiovascular disease risk and this elevated risk is only partly explained by the increased prevalence of traditional cardiovascular risk factors, such as age, gender, dyslipidaemia, hypertension, smoking, obesity, and diabetes mellitus (Nurmohamed et al. 2015). In addition, systemic inflammation and genetic factors also play a role (Nurmohamed et al. 2015). More recently, GC use has been directly related to an (dose-dependent) increase in cardiovascular death in RA (del Rincón et al. 2014). However, in this study, no effect on lipid profiles by different dosages was seen, as the factor low (<7.5 mg) versus moderate-to-high (>7.5 mg) dosage was excluded during confounder selection. A possible protective effect can be expected from concomitant use of hydroxychloroquine, which significantly lowered lysophospholipid scores in our study (decrease in mean lysophospholipid score = 0.180, 95 % CI (−0.347 to −0.013), *p* = 0.035; Table S5). It has also been reported to improve cholesterol levels, notably, in those treated with GCs (Hage et al. 2014).

Lysophospholipids, including lysophosphatidylcholine (LPC) and lysophosphatidylethanolamine (LPE), are an abundant lipid species, mainly functioning as transporters for free fatty acids. The difference between LPC and LPE is based only on the functional head group, respectively, choline or ethanolamine. The functions of LPEs are underreported, hampering their biological interpretation. Studies show that LPCs have properties resembling extracellular growth factors and signalling molecules (Ishii et al. 2004). In vivo, LPCs are generated from phospholipase A1/A2 catalysed hydrolysis of phosphatidylcholines, the basic component of membranes (Pruzanski et al. 2007). In addition, LPCs are released from phosphatidylcholines by the action of lecithin cholesterol acyltransferase in plasma (Kougias et al. 2006). Most of the circulating LPC is bound to albumin, but they are also a major component of lipoprotein particles, where they are a known constituent of oxidised low-density lipoproteins (LDL) (Chisolm and Chai 2000; Marathe et al. 2001), a well-known risk factor for cardiovascular diseases (Maiolino et al. 2013). The lysophospholipid-related gender differences are, therefore, potentially relevant with respect to the risk of cardiovascular events in RA patients, which could eventually guide the adjustment of treatment strategies for either males or females.

As patients were included in BiOCURA based on the necessity of biological treatment and not GC treatment, it was only possible to use samples of users and non-users, but not before and after GC initiation. Future studies are needed to validate our results, preferably before and after initiation of GC treatment. In addition, the role of lipid profiles (including triglycerides, diglycerides, and sphingomyelins) in the association between GC use, gender, and cardiovascular death should be clarified to fully understand and (specifically) prevent unwanted clinical (side)effects.

## Conclusion

After correcting for confounding factors, lysophosphatidylcholines and lysophosphatidylethanolamines in female RA patients with GC treatment were significantly higher than in female patients not taking GCs, whereas in male RA patients, these lysophospholipids levels were similar between GC users and non-users. These results could contribute to a better understanding and estimation of safety of GC drugs for male and female RA patients separately, particularly in relation to cardiovascular events.

## Electronic supplementary material

Below is the link to the electronic supplementary material.

Supplementary material 1 (PDF 228 kb)
