# Supplementary material for: Differences between serum polar lipid profiles of male and female rheumatoid arthritis patients in response to glucocorticoid treatment
Source: Inflammopharmacology. 2016 Sep 28;24(6):397–402. doi: 10.1007/s10787-016-0284-1 (PMC5119840; doi:10.1007/s10787-016-0284-1)
Supplement: Supplementary file 1 — Supplementary material 1 (PDF 228 kb) [file 10787_2016_284_MOESM1_ESM.pdf]

Electronic supplementary material

Journal: Inflammopharmacology

Differences between serum polar lipid profile of male and female rheumatoid arthritis patients in response to glucocorticoid treatment

Junzeng Fu *, Bart V.J. Cuppen, Paco M.J. Welsing, Herman van Wietmarschen, Amy C. Harms, Ruud Berger, Slavik Koval, Ruth D.E. Fritsch-Stork, Johannes W.J. Bijlsma, Thomas Hankemeier, Jan van der Greef, and Floris P.J.G. Lafeber

***Corresponding author:** E-mail: j.fu@lacdr.leidenuniv.nl;; Leiden Academic Center for Drug Research, Leiden University, Leiden, the Netherlands (J. Fu)

Method—lipidomics profiling

For the detection of polar lipids, each 20 µL serum was spiked with internal standard (ISTD) mix and lipids were extracted by 440 µL methanol. After centrifugation, the supernatant was transferred to an Eppendorf tube and the solvent was evaporated using a Speedvac. The dried lipids were reconstituted in 145 µL isopropanol, 0.1% formic acid and transferred to autosampler vials. In total 8 µL was injected into ultra-performance liquid chromatography coupled to electrospray ionization-quadrupole time-of-flight (Agilent 6530 San Jose,A, USA) with an ACQUITY UPLC™ HSS T3 column (1.8 µm, 2.1×100mm). Lipids were detected in full scan in the negative mode.

Figures

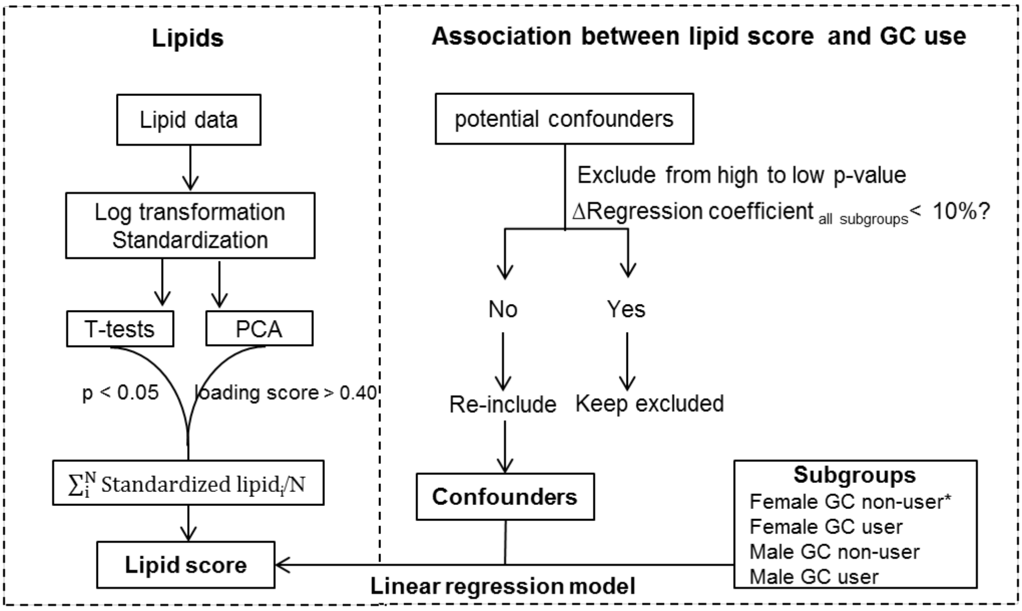

Fig S1. A schematic overview of the statistical analyses. * the subgroup “Female-GC non-user” is set as the reference group in the linear regression analysis.

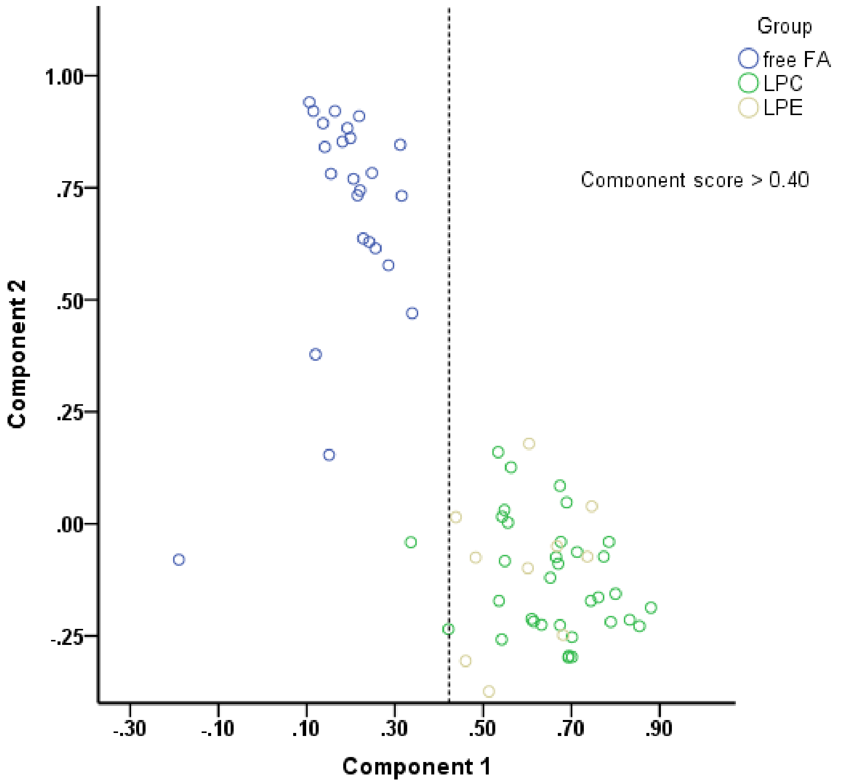

Fig. S2 Graphical representation of loading scores of 68 lipids in component 1 and 2 by PCA analysis. All the lysophospholipids (green and yellow circles) are with loading scores > 0.40 in component 1 (on the right side of the dash line). FA: fatty acid; LPC: lysophosphatidylcholines; LPE: lysophosphatidylethanolamines .

Table S1. Baseline characteristics of patients in the cohort (n=281), split for GC non-users and users, females and males.

**Item**	**Females**		**Males**		**p-value**
	**GC non-users (n = 136)**	**GC users (n = 77)**	**GC non-users (n = 26)**	**GC users (n = 42)**	
**Age, years, mean ± SD [a]**	53.2 ± 12.8	53.8 ± 14.1	54.2 ± 12.2	57.0 ± 11.7	0.428
**Body mass index, kg/m^2, mean ± SD [a]**	27.2 ± 5.8	27.0 ± 5.2	26.7 ± 3.4	25.0 ± 3.2	0.115
**Current smoker, n (%) [b]**	28 (20.6)	11 (14.3)	12 (46.2)	19 (45.2)	**<0.001**
**Positive RF, n (%) [b]**	91 (66.9)	38 (49.4)	15 (57.7)	34 (81.0)	**0.004**
**Positive ACPA, n (%) [b]**	90 (66.2)	49 (63.6)	17 (65.4)	31 (73.8)	0.726
**CRP, mg/dL, median (IQR) [c]**	6.0 (2.7-15.0)	5.0 (3.0-16.5)	5.5 (2.3-12.0)	10 (2.3-35.3)	0.487
**Use of other DMARDs, n (%) [b]**					
Methotrexate, n (%) [b]	99 (72.8)	48 (62.3)	22 (84.6)	32 (76.2)	0.115
Leflunomide, n (%) [b]	17 (12.5)	9 (11.7)	2 (7.7)	7 (16.7)	0.737
Hydroxychloroquine, n (%) [b]	21 (15.4)	22 (28.6)	9 (34.6)	10 (23.8)	0.050
**Use of non-DMARDs**					
NSAIDs, n (%) [b]	94 (69.1)	39 (50.6)	20 (76.9)	22 (52.4)	**0.010**
Anticoncepcion, n (%) [b]	10 (7.4)	5 (6.5)	0 (0.0)	0 (0.0)	0.162
Antidiabetics, n (%) [b]	5 (3.7)	4 (5.2)	1 (3.8)	1 (2.4)	0.892
Antihyperlipedemia, n (%) [b]	18 (13.2)	13 (16.9)	6 (23.1)	1 (2.4)	0.065
Bisphosphonates, n (%) [b]	34 (25.0)	58 (75.3)	3 (11.5)	30 (71.4)	**<0.001**
**DAS28, mean ± SD [a]**	4.6 ± 1.2	4.6 ± 1.2	3.9 ± 1.1	4.4 ± 1.4	0.071
TJC, median (IQR) [c]	8.0 (2.3-12.0)	7.0 (2.0-15.0)	4.0 (1.0-6.3)	6.0 (0.0-14.0)	0.069
SJC, median (IQR) [c]	2.0 (0.0-4.0)	2.0 (0.0-4.0)	1.0 (0.0-3.0)	1.0 (0.0-4.3)	0.885
VAS-GH, mean ± SD [a]	58.6 ± 24.2	56.4 ± 22.7	49.2 ± 25.1	57.1 ± 23.3	0.324
ESR, mm/h, median (IQR) [c]	19.0 (11.0-37.0)	22.0 (6.5-39.0)	15.5 (6.0-34.3)	19.0 (9.5-47.5)	0.786

P-values for comparisons among four subgroups were calculated by one-way ANOVA [a], Chi-square tests [b], and Kruskal wallis H tests [c]) based on distribution of the clinical parameter. Bold values indicate significant p-values ($p < 0.05$).

ACPA: anti citrullinated protein antibody,RP: c-reactive protein, DAS28: disease activity score based on a 28-joint count, DMARDs: disease-modifying antirheumatic drugs, ESR: erythrocyte sedimentation rate, IQR: interquartile range, NASID: non-steroid anti-inflammatory drug, RF: rheumatoid factor, SD: standard deviation, SJC: swollen joint count, TJC: tender joint count, VAS-GH: visual analogue scale-general health.

Table S2. List of detected metabolites in lipidomics profiling

Lipid Class	Lipid Maps	Metabolite species	Amount (n)
Free fatty acids (FA)	FA01	C14:0, C14:1, C15:0, C16:0, C16:1, C17:0, C17:1,C18:0, C18:1, C18:2, C18:3-ω3/ω6, C20:0, C20:1, C20:2, C20:3-ω3/ω6, C20:3-ω9, C20:4-ω6, C20:5-ω3, C22:4, C22:5-ω3,C 22:5-ω6, C22:6, C24:0, C24:1	24
Lysophosphatidylcholine (LPC)	GP0105	*sn1:* C14:0, C15:0, C16:0, C16:1, C18:0, C18:1, C18:2, C18:3-ω3/ω6, C19:0, C20:1, C20:2, C20:3-ω3/ω6, C20:3-ω9, C20:4, C20:5, C22:4, C22:5-ω3, C22:5-ω6, C22:6<br>*sn2:* C14:0, C16:0, C16:1, C18:0, C18:1, C18:2, C18:3-ω3/ω6, C20:3-ω3/ω6,C 20:4, C20:5, C22:6	30
Lysophosphatidylethanolamine (LPE)	GP0205	C16:0, C18:0, C18:1, C18:2, C20:3-ω3/ω6, C20:4, C20:5, C22:5-ω3, C22:5-ω6, C22:6	10
Plasmalogen Lysophosphatidylcholine (LPC-O) [a]	GP0106	C16:0; C18:0; C18:1; C18:2	4

The "*sn*1-” or “*sn*2-” prefix is used to indicate the position of the fatty acid chain esterified to the glycerol backbone of a lysophosphatidylcholine.

[a] The 'LPC-O' is used to identify a plasmalogen lysophosphatidylcholine, where the fatty acid chain is attached via a vinyl ether linkage to the glycerol backbone. The ‘ω3’, ‘ω6’, ‘ω9’ are used to indicate double bond (C=C) at the third, sixed or ninth carbon atom from the end of the fatty acid chain.

Table S3. Differences in 68 serum lipid levels between GC users and non-users in males and females, respectively. The mean differences between GC non-users and users are shown. *P*-values were calculated by means of an independent sample t-test.

Metabolite name	Males (GC user vs. GC non-user)		Females (GC user vs. GC non-user)	
	$Mean_{non-user}$-$Mean_{user}$	p value	$Mean_{non-user}$-$Mean_{user}$	p value
sn1-LPC (14:0)	-0.106	0.602	-0.444	0.001
sn1-LPC (15:0)	-0.116	0.633	-0.346	0.010
sn1-LPC (16:0)	0.027	0.796	-0.301	<0.001
sn1-LPC (16:1)	-0.181	0.422	-0.507	<0.001
sn1-LPC (18:0)	0.289	0.122	-0.148	0.304
sn1-LPC (18:1)	-0.091	0.512	-0.368	<0.001
sn1-LPC (18:2)	-0.283	0.027	-0.332	<0.001
sn1-LPC (18:3-ω3/ω6)	-0.343	0.077	-0.393	0.004
sn1-LPC (19:0)	0.252	0.153	0.091	0.494
sn1-LPC (20:1)	0.120	0.568	-0.207	0.099
sn1-LPC (20:2)	-0.153	0.408	-0.395	0.004
sn1-LPC (20:3-ω3/ω6)	0.152	0.342	-0.226	0.054
sn1-LPC (20:3-ω9)	0.284	0.276	-0.198	0.144
sn1-LPC (20:4)	0.183	0.244	-0.2	0.032
sn1-LPC (20:5)	-0.417	0.062	-0.237	0.074
sn1-LPC (22:4)	0.228	0.359	-0.283	0.047
sn1-LPC (22:5-ω3)	-0.161	0.369	-0.373	0.007
sn1-LPC (22:5-ω6)	0.357	0.128	-0.099	0.455
sn1-LPC (22:6)	0.107	0.610	-0.021	0.882
sn2-LPC (14:0)	-0.157	0.405	-0.427	0.001
sn2-LPC (16:0)	0.178	0.341	-0.501	<0.001
sn2-LPC (16:1)	-0.237	0.308	-0.487	<0.001
sn2-LPC (18:0)	0.211	0.098	-0.115	0.231
sn2-LPC (18:1)	-0.114	0.559	-0.443	<0.001
sn2-LPC (18:2)	-0.395	0.052	-0.533	<0.001
sn2-LPC (18:3-ω3/ω6)	-0.362	0.161	-0.411	0.001
sn2-LPC (20:3-ω3/ω6)	0.226	0.281	-0.263	0.062
sn2-LPC (20:4)	0.215	0.384	-0.331	0.021
sn2-LPC (20:5)	-0.421	0.075	-0.278	0.046
sn2-LPC (22:6)	0.092	0.666	-0.07	0.616
LPC (O-16:0)	0.157	0.466	-0.343	0.020
LPC (O-18:0)	-0.019	0.928	-0.419	0.003
LPC (O-18:1)	0.190	0.410	-0.152	0.299
LPC (O-18:2)	0.080	0.707	-0.398	0.004
LPE (16:0)	-0.279	0.143	-0.505	0.001
LPE (18:0)	-0.102	0.588	-0.587	<0.001
LPE (18:1)	0.031	0.883	-0.449	0.001
LPE (18:2)	-0.349	0.093	-0.497	<0.001
LPE (20:3-ω3/ω6)	-0.051	0.789	-0.469	0.001
LPE (20:4)	-0.062	0.785	-0.685	<0.001
LPE (20:5)	-0.463	0.029	-0.352	0.012
LPE (22:5-ω3)	-0.328	0.089	-0.694	<0.001

LPE (22:5-ω6)	0.294	0.215	-0.356	0.009
LPE (22:6)	-0.242	0.218	-0.512	0.001
FA (14:0)	0.089	0.654	-0.014	0.924
FA (14:1)	0.038	0.865	0.159	0.275
FA (15:0)	0.129	0.517	-0.027	0.855
FA (16:0)	-0.083	0.680	-0.006	0.966
FA (16:1)	0.113	0.600	0.042	0.770
FA (17:0)	-0.097	0.652	-0.057	0.692
FA (17:1)	0.041	0.852	0.023	0.872
FA (18:0)	-0.072	0.725	0.109	0.456
FA (18:1)	0.134	0.551	0.075	0.609
FA (18:2)	0.089	0.689	0.035	0.805
FA (18:3-ω3/ω6)	0.190	0.364	0.009	0.950
FA (20:0)	0.039	0.878	-0.071	0.620
FA (20:1)	0.054	0.803	-0.013	0.925
FA (20:2)	0.011	0.969	-0.042	0.761
FA (20:3-ω3/ω6)	0.377	0.109	-0.107	0.445
FA (20:3-ω9)	0.500	0.021	-0.074	0.595
FA (20:4-ω6)	0.250	0.212	-0.013	0.905
FA (20:5-ω3)	-0.105	0.628	-0.098	0.463
FA (22:4)	0.237	0.234	-0.077	0.543
FA (22:5-ω3)	-0.032	0.866	-0.143	0.271
FA (22:5-ω6)	0.247	0.261	0.017	0.901
FA (22:6)	0.371	0.107	0.072	0.585
FA (24:0)	0.071	0.783	0.028	0.838
FA (24:1)	-0.013	0.951	0.016	0.901

Table S4. Loading scores of 68 lipids in principle components analysis. Component loading scores of all lipids were presented for the first five components (with eigenvalue >1). Since the LPCs and LPEs showed high component loadings in one component (the first) and only with positive values, they all highly correlate in the same direction. Considering all LPCs and LPEs significantly different between GC users and non-users, all loading scores were ≥0.438.

Metabolite name	Component				
	1	2	3	4	5
sn1*-LPC (14:0)	0.701	-0.253	0.057	0.147	-0.357
sn1*-LPC (15:0)	0.610	-0.212	-0.159	0.053	-0.066
sn1*-LPC (16:0)	0.880	-0.187	-0.077	-0.080	-0.009
sn1*-LPC (16:1)	0.773	-0.073	0.152	0.044	-0.319
sn1*-LPC (18:0)	0.744	-0.172	-0.249	-0.082	0.295
sn1*-LPC (18:1)	0.854	-0.228	0.023	0.131	0.135
sn1*-LPC (18:2)	0.701	-0.297	-0.036	0.181	0.429
sn1*-LPC (18:3-ω3/ω6)	0.694	-0.295	0.047	0.344	-0.100
***sn1*-LPC (19:0)**	0.421	-0.235	-0.315	-0.029	0.291
***sn1*-LPC (20:1)**	0.336	-0.041	-0.252	-0.132	0.203
sn1*-LPC (20:2)	0.800	-0.156	0.126	-0.108	0.108
***sn1*-LPC (20:3-ω3/ω6)**	0.665	-0.074	0.404	-0.312	-0.133
***sn1*-LPC (20:3-ω9)**	0.652	-0.120	0.345	-0.165	-0.213
sn1*-LPC (20:4)	0.674	0.085	0.154	-0.492	0.032
***sn1*-LPC (20:5)**	0.543	0.016	-0.668	0.069	-0.296
sn1*-LPC (22:4)	0.676	-0.040	0.539	-0.326	0.026
sn1*-LPC (22:5-ω3)	0.785	-0.040	-0.219	-0.022	0.014
***sn1*-LPC (22:5-ω6)**	0.556	0.003	0.569	-0.405	-0.068
***sn1*-LPC (22:6)**	0.534	0.160	-0.574	-0.254	-0.084
sn2*-LPC (14:0)	0.674	-0.226	0.057	0.173	-0.343
sn2*-LPC (16:0)	0.832	-0.214	-0.050	-0.057	-0.051
sn2*-LPC (16:1)	0.713	-0.063	0.183	0.026	-0.314
***sn2*-LPC (18:0)**	0.761	-0.164	-0.235	-0.079	0.283
sn2*-LPC (18:1)	0.789	-0.219	0.037	0.112	0.121
sn2*-LPC (18:2)	0.693	-0.298	-0.018	0.160	0.439
***sn2*-LPC (18:3-ω3/ω6) ***	0.614	-0.218	0.049	0.322	-0.104
***sn2*-LPC (20:3-ω3/ω6)**	0.670	-0.089	0.385	-0.302	-0.079
sn2*-LPC (20:4)	0.689	0.048	0.175	-0.463	0.024
sn2*-LPC (20:5)	0.548	0.031	-0.643	0.089	-0.315
***sn2*-LPC (22:6)**	0.563	0.126	-0.551	-0.251	-0.070
**LPC (O-16:0)***	0.632	-0.225	-0.219	-0.301	0.408
**LPC (O-18:0)***	0.542	-0.258	-0.252	-0.242	0.411
**LPC (O-18:1)***	0.536	-0.172	-0.241	-0.290	0.544
**LPC (O-18:2)***	0.549	-0.083	-0.283	-0.209	0.406
**LPE (16:0)***	0.681	-0.248	0.066	0.176	-0.278
**LPE (18:0)***	0.746	0.039	0.082	0.184	-0.072
**LPE (18:1)***	0.460	-0.306	0.210	0.558	0.173
**LPE (18:2)***	0.513	-0.374	0.219	0.588	0.217
**LPE (20:3-ω3/ω6)***	0.601	-0.099	0.512	0.237	-0.167

**LPE (20:4)***	0.667	-0.050	0.375	0.126	0.044
**LPE (20:5)***	0.483	-0.075	-0.496	0.458	-0.328
**LPE (22:5-ω3)***	0.736	-0.073	0.161	0.340	-0.156
**LPE (22:5-ω6)***	0.438	0.015	0.672	-0.080	-0.177
**LPE (22:6)***	0.604	0.179	-0.323	0.128	-0.235
**FA (14:0)**	0.192	0.883	0.066	0.221	-0.006
**FA (14:1)**	0.141	0.841	0.023	0.089	-0.061
**FA (15:0)**	0.215	0.733	0.102	0.210	0.065
**FA (16:0)**	0.206	0.770	0.190	0.294	0.183
**FA (16:1)**	0.164	0.921	0.067	0.020	0.000
**FA (17:0)**	0.221	0.744	0.069	0.239	0.151
**FA (17:1)**	0.219	0.910	0.031	0.068	0.043
**FA (18:0)**	0.120	0.378	0.218	0.392	0.259
**FA (18:1)**	0.106	0.941	0.057	0.073	0.130
**FA (18:2)**	0.137	0.894	0.012	0.087	0.206
**FA (18:3-ω3/ω6)**	0.181	0.853	-0.044	0.183	0.119
**FA (20:0)**	0.151	0.154	0.105	0.336	0.209
**FA (20:1)**	0.115	0.921	-0.029	0.071	0.160
**FA (20:2)**	0.155	0.781	0.008	-0.129	0.112
**FA (20:3-ω3/ω6)**	0.315	0.732	0.216	-0.184	-0.111
**FA (20:3-ω9)***	0.285	0.577	0.152	-0.208	-0.124
**FA (20:4-ω6)**	0.256	0.615	-0.019	-0.349	0.074
**FA (20:5-ω3)**	0.339	0.470	-0.686	0.085	-0.244
**FA (22:4)**	0.199	0.861	0.266	-0.164	-0.002
**FA (22:5-ω3)**	0.312	0.846	-0.275	0.076	-0.065
**FA (22:5-ω6)**	0.248	0.783	0.271	-0.237	-0.142
**FA (22:6)**	0.242	0.629	-0.494	-0.076	-0.163
**FA (24:0)**	-0.190	-0.080	0.319	0.392	0.553
**FA (24:1)**	0.228	0.637	-0.163	0.125	0.118

* Metabolites were also significantly different in independent t-tests between GC users and non-users, in either females or males (Figure 1, Table S3).

Table S5. The final regression model investigating the association between gender and GC use on the lysophospholipid score.

Variables	Coefficients [a]	95%-confidence interval	p value
**Male patients no GC use (reference group)**	0.149	(-0.491 to 0.789)	0.649
**Male patients GC use**	0.122	(-0.160 to 0.405)	0.397
**Female patients no GC use**	-0.310	(-0.604 to -0.015)	0.041
**Female patients GC use**	0.089	(-0.212 to 0.390)	0.565
**Age**	0.001	(-0.008 to 0.007)	0.937
**Menopausal status**	0.286	(0.053 to 0.519)	0.017
**BMI (kg/m^2)**	0.006	(-0.009 to 0.020)	0.447
**RF positivity**	-0.247	(-0.432 to -0.063)	0.009
**ACPA positivity**	0.182	(-0.008 to 0.372)	0.061
**CRP (log transformed, mg/dL)**	-0.122	(-0.192 to -0.052)	0.001
**ESR (log transformed, mm/h)**	0.058	(-0.031 to 0.147)	0.202
**DAS28**	-0.014	(-0.078 to 0.050)	0.663
**Concomitant methotrexate**	-0.023	(-0.181 to 0.134)	0.773
**Concomitant hydroxychloroquin**	-0.180	(-0.347 to -0.013)	0.035
**Antidiabetics**	-0.132	(-0.493 to 0.229)	0.474
**NSAIDs**	-0.068	(-0.215 to 0.078)	0.362

[a] Coefficients indicate the changes in mean lysophospholipid score after correcting for confounders—BMI, menopausal status, RF positive, log-transformed CRP, log transformed ESR, DAS28, and concomitant drugs (methotrexate, hydroxychloroquine, anti-diabetic drug and NSAIDs). BMI: body mass index; CRP: C-reactive protein; GC: glucocorticoid; RF: rheumatoid factor; ACPA: anti citrullinated protein antibody; ESR: erythrocyte sedimentation rate; DAS28: disease activity score based on a 28-joint count; NSAIDs: non-steroid anti-inflammatory drugs
